# Prevalence of *Borrelia* spp. and *Rickettsia* spp. in *Ixodes ricinus* Occurring in Suboptimal Meadow Habitats in Eastern Poland

**DOI:** 10.3390/pathogens15070722

**Published:** 2026-07-09

**Authors:** Joanna Kulisz, Zbigniew Zając, Aneta Woźniak, Sara Moutailler, Angélique Foucault-Simonin, Alejandro Cabezas-Cruz

**Affiliations:** 1 Department of Biology and Parasitology, Medical University of Lublin, Radziwiłłowska 11, 20-080 Lublin, Polandaneta.wozniak@umlub.edu.pl (A.W.); 2 Laboratoire de Santé Animale, UMR BIPAR, Ecole Nationale Vétérinaire d’Alfort, INRAE, Anses, 94700 Maisons-Alfort, France; sara.moutailler@anses.fr (S.M.); angelique.foucaultsimonin@anses.fr (A.F.-S.); alejandro.cabezas@vet-alfort.fr (A.C.-C.)

**Keywords:** tick-borne pathogens, ticks, *Ixodes ricinus*, *Borrelia* spp., *Rickettsia* spp., suboptimal meadow sites

## Abstract

*Ixodes ricinus* is the principal vector of numerous tick-borne pathogens (TBPs) in Europe and is typically associated with forest sites that provide favorable microclimatic conditions. However, this species may also occur in meadow ecosystems, which are generally regarded as suboptimal environments and remain insufficiently studied from an epidemiological perspective. The aim of this study was to determine and compare the prevalence of *Borrelia* spp. and *Rickettsia* spp. in adult *I. ricinus* occurring in urban and rural meadow sites in eastern Poland. Ticks collected between June 2023 and May 2024 were screened for *Borrelia* spp. and *Rickettsia* spp. using high-throughput microfluidic real-time PCR targeting the 23S rRNA and ITS regions, respectively. The DNA of *Borrelia* spp. was detected in 14.8% of ticks from the urban site and 10.7% from the rural site, whereas *Rickettsia* spp. were detected in 5.6% and 8.9% of specimens, respectively. No significant differences in pathogen prevalence were observed between sites. The results confirmed the presence of *Borrelia* spp. and *Rickettsia* spp. in adult *I. ricinus* collected in urban and rural meadow sites.

## 1. Introduction

Tick-borne diseases (TBDs) represent an increasing public health challenge in Europe due to the expanding distribution of ticks and the growing incidence of tick-borne infections [1]. Among European tick species, *Ixodes ricinus* is the most important vector of pathogens affecting humans and animals, including spirochetes of the *Borrelia burgdorferi* sensu lato complex and spotted fever group *Rickettsia* spp. These microorganisms are widely distributed throughout Europe and are responsible for a substantial proportion of tick-borne bacterial infections [2].

The occurrence and prevalence of tick-borne pathogens (TBPs) in *I. ricinus* are strongly influenced by ecological factors, including habitat characteristics, host community composition, and local environmental conditions [3]. Although this tick species is primarily associated with humid forested habitats that provide favorable microclimatic conditions, it can also persist in open habitats such as meadows, where suitable vertebrate hosts are available [4,5,6]. However, meadow ecosystems are generally regarded as suboptimal habitats for *I. ricinus* and consequently have received considerably less attention than forest environments in epidemiological studies [7,8].

Although eastern Poland is among the most extensively investigated regions in the country with respect to tick occurrence and TBPs circulation, data on the prevalence of *Borrelia* spp. and *Rickettsia* spp. in *I. ricinus* populations inhabiting meadow ecosystems remain limited [8,9,10]. This knowledge gap is especially evident for meadow habitats located within urbanized landscapes, where environmental conditions and host assemblages may differ substantially from those occurring in rural areas.

Urbanization can alter tick–host–pathogen relationships through changes in habitat structure, biodiversity, and vertebrate host communities, potentially affecting pathogen circulation within local tick populations [11,12]. Consequently, comparisons between urban and rural meadow habitats may provide valuable insights into the epidemiology of TBPs in environments that are not typically considered optimal for *I. ricinus*.

Therefore, the aim of this preliminary study was to determine and compare the prevalence of *Borrelia* spp. and *Rickettsia* spp. in adult *I. ricinus* ticks collected from urban and rural meadow sites in eastern Poland to evaluate whether these suboptimal site types support pathogen circulation.

## 2. Materials and Methods

### 2.1. Study Area and Tick Surveillance

**Figure 1:**
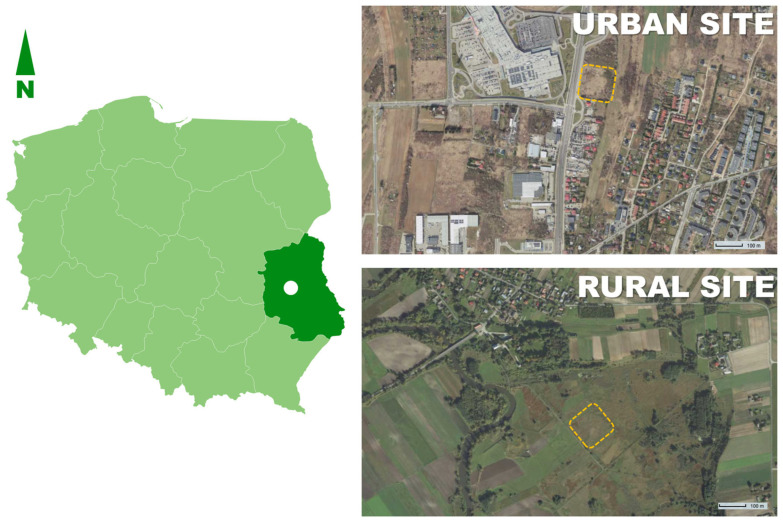
Location of the study sites (urban and rural) within the Lublin Voivodeship and Poland. The map shows the geographical distribution (white dot) of the investigated urban (51°16′52.9″ N 22°34′32.2″ E) and rural (51°21′15.6″ N 22°45′39.6″ E) study sites in relation to the Lublin Voivodeship (dark green) and the territory of Poland. The investigated areas are indicated by a yellow dashed line. The figure was created with Datawrapper (https://www.datawrapper.de/; accessed on 5 May 2026) and Geoportal2.pl (https://www.geoportal2.pl/pl/; accessed on 5 May 2026).

The study was conducted in eastern Poland, a region characterized by a temperate transitional climate [13]. Tick sampling was performed between June 2023 and May 2024 at two-week intervals, encompassing the seasonal activity period of adult *I. ricinus* in this region [10]. Ticks were collected from two 900 m^2^ meadow sites representing urban (51°16′52.9″ N 22°34′32.2″ E) and rural (51°21′15.6″ N 22°45′39.6″ E) landscapes that were separated by an approximate straight-line distance of 15 km (Figure 1). Therefore, throughout this manuscript, the study sites are referred to as the urban and rural sites, respectively. Both sites were dominated by dense grass vegetation and showed evidence of the presence of potential tick hosts, including small mammals and wild boars.

Ticks were collected using the standard flagging method. A detailed description of the sampling design and collection procedure is provided in our previous studies [8,10].

Following collection, ticks were transported to the laboratory and identified according to species, sex, and developmental stage using the identification key [14]. All specimens were subsequently stored at −20 °C until molecular analyses were performed.

### 2.2. Molecular Analysis

#### 2.2.1. DNA Extraction and Preamplification

During the study period, a total of 247 adult *I. ricinus* ticks were collected, including 176 specimens from the urban site and 71 from the rural site. Owing to the unequal abundance of ticks between study sites, a subset of 110 adults (54 from the urban site and 56 from the rural site) was selected for DNA extraction and subsequent molecular analyses to ensure comparable sample sizes between sites. Because the studied meadow sites were characterized by dense, tall grass vegetation, only a limited number of *I. ricinus* nymphs were collected during the sampling period. Therefore, nymphs were not included in the subsequent molecular analyses.

In the first step ticks were rinsed with distilled water, air-dried, and dissected into smaller fragments using a sterile scalpel. Genomic DNA from tick specimens was extracted using the Genomic Mini AX Tissue kit (A&A Biotechnology, Gdynia, Poland) according to the manufacturer’s protocol. Next, DNA concentration and purity were assessed spectrophotometrically using a NanoDrop 2000 instrument (Thermo Scientific, Waltham, MA, USA) by measuring absorbance at 260/280 nm. DNA concentrations ranged from 15 to 300 ng/µL, with a final elution volume of 35 µL. All extracted DNA samples were stored at −20 °C until further molecular analyses.

To increase the DNA amount, preamplification was performed using the PreAmp Master Mix kit (Standard Biotools, San Francisco, CA, USA) according to the manufacturer’s instructions. All primer pairs targeting TBP were pooled in equal volumes to obtain a final concentration of 0.2 µM for each primer pair [15].

Each preamplification reaction was prepared in a final volume of 5 µL containing 1 µL of Perfecta Preamp 5× reaction buffer, 1.25 µL of the pooled primer mix, 1.5 µL of distilled water, and 1.25 µL of the DNA template. Thermal cycling conditions consisted of an initial denaturation step at 95 °C for 2 min, followed by 14 cycles of denaturation at 95 °C for 15 s and annealing/extension at 60 °C for 4 min.

Following amplification, the products were diluted 1:10 with 45 µL of Milli-Q ultrapure water and stored at −20 °C until further analysis.

#### 2.2.2. Microfluidic Real-Time PCR for High-Throughput Detection of Pathogens

High-throughput detection of tick-borne microorganisms was performed using the BioMark™ real-time PCR system (Standard Biotools, San Francisco, CA, USA). Real-time PCR reactions were carried out using FAM-labeled and BHQ1-labeled TaqMan probes in combination with TaqMan Gene Expression Master Mix according to the manufacturer’s instructions (Applied Biosystems, Les Ulis, France). Thermal cycling conditions consisted of an initial incubation reaction at 50 °C for 2 min, followed by enzyme activation at 95 °C for 10 min, 40 cycles of denaturation at 95 °C for 15 s and annealing/extension at 60 °C for 1 min.

Detection of *Borrelia* spp. was based on amplification of the 23S rRNA gene using the primer pair F: GAGTCTTAAAAGGGCGATTTAGT and R: CTTCAGCCTGGCCATAAATAG, whereas *Rickettsia* spp. was detected by amplification of the ITS region using the primer pair F: TTTGAAGGAGACACGGAACACA and R: TCCGGTACTCAAATCCTCACGTA. To verify the performance of the assay, the *eae* gene of *Escherichia coli* (F:CATTGATCAGGATTTTTCTGGTGAT and R: R-CTCATGCGGAAATAGCCGTTA) was used as an internal positive amplification control [15]. Nuclease-free water was used as a negative control.

#### 2.2.3. Confirmation of Microfluidic Real-Time PCR

To validate the results obtained by high-throughput real-time PCR, a subset of randomly selected positive samples was subjected to conventional PCR. For *Borrelia* spp., a fragment of the *flaB* gene was amplified using the primers F: GGAGCAAATCAAGATGAAGCAAT and R: TGAGCACCCTCTTGAACAGG. For *Rickettsia* spp., amplification targeted the *gltA* gene using the primers F: GTCGCAAATGTTCACGGTACTT and R: TCTTCGTGCATTTCTTTCCATTG [15]. PCR products were visualized by electrophoresis in agarose gels and examined under UV illumination.

### 2.3. Statistical Analysis

Differences in the prevalence of *Borrelia* spp. and *Rickettsia* spp. between urban and rural meadow sites were assessed separately using Fisher’s exact test. For each pathogen, 2 × 2 contingency tables were constructed based on the number of positive and negative *I. ricinus* specimens in each site type. Statistical significance was set at *p* < 0.05. Calculations were performed using GraphPad 8.4 (GraphPad Software Inc., La Jolla, CA, USA).

For each prevalence estimate, 95% confidence intervals (95% CIs) were calculated using the exact binomial Clopper–Pearson method implemented in the online Epitools epidemiological calculator (epitools.ausvet.com.au; accessed 21 June 2026).

## 3. Results

### Prevalence of Tick-Borne Pathogens

In the urban site, the DNA of *Borrelia* spp. was detected in 14.8% (95% CI: 6.6–27.1) of the analyzed ticks, whereas that of *Rickettsia* spp. was identified in 5.6% (95% CI: 1.2–15.4) of specimens. In the rural site, the prevalence of *Borrelia* spp. and *Rickettsia* spp. reached 10.7% (95% CI: 4.0–21.9) and 8.9% (95% CI: 3.0–19.6), respectively (Table 1). The prevalence of both pathogens was generally comparable between female and male ticks within each site, although in the urban meadow site *Borrelia* spp. and *Rickettsia* spp. were detected more frequently in females than in males; however, the absolute number of positive samples was low (Table 1).

Although *Borrelia* spp. were more frequently detected in ticks collected from the urban meadow and *Rickettsia* spp. were more frequently detected in those from the rural meadow, neither difference was statistically significant. The prevalence of *Borrelia* spp. did not differ significantly between sites (*p* = 0.577), nor did the prevalence of *Rickettsia* spp. (*p* = 0.716).

## 4. Discussion

The occurrence of *I. ricinus* is strongly determined by habitat characteristics, particularly those affecting microclimatic conditions [16]. This species is mostly associated with forest ecosystems, especially mature deciduous and mixed forests characterized by dense understory vegetation and a well-developed litter layer [10]. Such environments provide relatively stable temperatures and, most importantly, high humidity levels, which are essential for tick survival and development [10,16,17]. Among abiotic factors, humidity is considered one of the most important determinants of *I. ricinus* distribution because this species is particularly susceptible to water loss during off-host periods. Consequently, habitats that reduce the risk of desiccation are generally regarded as optimal for maintaining abundant tick populations [6,18].

In contrast, open habitats such as meadows are typically characterized by greater fluctuations in temperature and humidity, resulting in less favorable conditions for tick survival [8,10,19]. Nevertheless, the presence of sufficiently dense vegetation may partly compensate for these limitations by creating a more suitable microclimate near the ground surface and providing questing sites for host-seeking ticks. The meadow sites investigated in the present study (Figure 1) were dominated by dense grass vegetation, which likely contributed to the persistence of *I. ricinus* populations despite the generally suboptimal nature of these environments. Although such habitats cannot be considered optimal for this species, our findings confirm that they can support local tick populations and consequently maintain the circulation of medically important TBPs (Table 1).

The circulation of TBPs in local tick populations is not determined solely by abiotic habitat conditions, but also strongly depends on the availability, density, and composition of vertebrate hosts. Different host groups, including small mammals, birds, deer, and wild boars, may contribute differently to the maintenance of tick populations and to the circulation of particular microorganisms. Therefore, differences in the structure of local host communities may influence the prevalence of *Borrelia* spp. and *Rickettsia* spp. in questing *I. ricinus* ticks [5,8,12,18].

In this study, the presence of potential tick hosts was recorded only indirectly, based on field observations and signs of animal activity, including evidence of cervids, small mammals and wild boars. However, no systematic assessment of host density or host community composition was performed. Consequently, the observed differences in pathogen prevalence between the urban and rural meadow sites (Table 1) should be interpreted descriptively and cannot be mechanistically attributed to differences in host communities. Future studies on TBP circulation in meadow habitats should include structured host surveys or quantitative indicators of vertebrate host activity to clarify the ecological mechanisms underlying pathogen maintenance in these environments.

In the present study, the DNA of *Borrelia* spp. was detected in both investigated meadow sites, with a slightly higher prevalence in ticks collected from the urban site than from the rural site (14.8% and 10.7%, respectively) (Table 1). Although this difference was not statistically significant, the detection of *Borrelia* spp. in adult *I. ricinus* from both sites confirms that meadow ecosystems, despite being suboptimal for this tick species, may participate in the circulation of pathogens of major public health relevance.

Previous studies conducted in Europe have demonstrated considerable variability in the prevalence of *Borrelia* spp. in *I. ricinus* inhabiting meadow ecosystems. Depending on the geographical location, habitat characteristics, and host community composition, reported prevalence values have ranged from less than 1.0% to approximately 20.0% [20,21,22]. The prevalence of *Borrelia* spp. observed in the present study was lower than that reported in our previous investigation from the Lublin region, where the DNA of *Borrelia* spp. was detected in approximately 20.0% of *I. ricinus* collected from meadow ecosystems [8]. This difference may result from site-specific ecological conditions, as well as from the limited sample size of the present preliminary study. Therefore, these findings should not be interpreted as directly equivalent, but rather as further evidence that meadow environments in eastern Poland may support the circulation of *Borrelia* spp., although prevalence may vary substantially between individual sites.

*Rickettsia* spp. were also detected in ticks from both study sites, with prevalence reaching 5.6% in the urban site and 8.9% in the rural site (Table 1). Although the epidemiological significance of *Rickettsia* spp. is generally lower than that of *Borrelia* spp., these bacteria should not be underestimated, particularly because several species transmitted by ticks belong to the spotted fever group and may cause human rickettsioses [23]. *Rickettsia* spp. are also frequently reported in *I. ricinus* populations throughout Europe, including ticks collected from meadow sites. Previous studies have demonstrated substantial geographical variation in infection rates, with prevalence exceeding up to 50.0% [24,25].

A limitation of the present study is that the applied molecular assays allowed the detection of the DNA of *Borrelia* spp. and *Rickettsia* spp. at the genus level only, without species- or genospecies-level identification. However, given the preliminary character of this study, the detection of these microorganisms in *I. ricinus* from the investigated meadow sites still provides epidemiologically relevant evidence that such habitats may participate in the circulation of TBPs. Future studies should include sequencing or species-specific assays to determine the exact pathogen composition and to better assess the clinical relevance of these findings. Moreover, potential seasonal variation in environmental conditions, including temperature and relative humidity, was not analyzed in the present study. These variables constitute part of a broader research project conducted at the same study sites and will be examined in detail in a separate publication. Future analyses will specifically address seasonal dynamics and their potential influence on tick activity and pathogen occurrence. Another limitation of this study is that it was not specifically designed to assess sex-related differences in pathogen prevalence. Although *Borrelia* spp. and *Rickettsia* spp. were detected more frequently in female ticks at the urban site, the low number of positive samples precludes drawing firm conclusions. Future studies with larger sample sizes are needed to verify whether tick sex influences pathogen prevalence.

## 5. Conclusions

The present study demonstrated the occurrence of *Borrelia* spp. and *Rickettsia* spp. in *Ixodes ricinus* inhabiting urban and rural meadow sites in eastern Poland. Although these environments are generally regarded as suboptimal for this tick species, both pathogens were detected in ticks collected from each study site. However, due to the preliminary character of the study, the limited number of sampling sites and analyzed ticks, and genus-level pathogen detection, the results should be interpreted cautiously and should not be generalized to all urban and rural meadow habitats. Future studies should include replicated meadow sites, larger sample sizes, quantitative assessment of vertebrate host communities, seasonal analyses, and species- or genospecies-level identification of detected pathogens.

## Figures and Tables

**Table 1:** Prevalence of *Borrelia* spp. and *Rickettsia* spp. in adult *Ixodes ricinus* collected from urban and rural meadow sites in eastern Poland.

TBP	Number of Positive Samples and Prevalence (%); [95% CI]					
	Study Site					
	Urban			Rural		
	F (*n* = 28)	M (*n* = 26)	T (*n* = 54)	F (*n* = 29)	M (*n* = 27)	T (*n* = 56)
*Borrelia* spp.	5 (17.90) [6.1–36.9]	3 (11.5) [2.4–30.2]	8 (14.8) [6.6–27.1]	3 (10.3) [2.2–27.4]	3 (11.1) [2.4–29.2]	6 (10.7) [4.0–21.9]
*Rickettsia* spp.	2 (7.1) [0.9–23.5]	1 (3.8) [0.1–19.6]	3 (5.6) [1.2–15.4]	2 (6.9) [0.8–22.8]	3 (11.1) [2.4–29.2]	5 (8.9) [3.0–19.6]

TBP: tick-borne pathogen, F: female, M: male, T: total, *n*: number of examined specimens, CI: confidence interval.

## Data Availability

All data generated in this study is included within the text of the manuscript.

## Abbreviations

The following abbreviations are used in this manuscript:

TBDs	Tick-borne diseases
TBPs	Tick-borne pathogens
F	Female
M	Male
CI	Confidence interval
